# Reovirus Nonstructural Protein σNS Recruits Viral RNA to Replication Organelles

**DOI:** 10.1128/mBio.01408-21

**Published:** 2021-07-06

**Authors:** Christopher H. Lee, Krishnan Raghunathan, Gwen M. Taylor, Andrea J. French, Raquel Tenorio, Isabel Fernández de Castro, Cristina Risco, John S. L. Parker, Terence S. Dermody

**Affiliations:** a Department of Microbiology and Molecular Genetics, University of Pittsburgh School of Medicine, Pittsburgh, Pennsylvania, USA; b Institute of Infection, Inflammation, and Immunity, University of Pittsburgh Medical Center Children’s Hospital of Pittsburgh, Pittsburgh, Pennsylvania, USA; c Department of Pediatrics, University of Pittsburgh School of Medicine, Pittsburgh, Pennsylvania, USA; d Cell Structure Laboratory, National Center for Biotechnology, CNB-CSIC, Madrid, Spain; e Baker Institute for Animal Health, College of Veterinary Medicine, Cornell University, Ithaca, New York, USA; Duke University Medical Center

**Keywords:** reovirus, σNS, μNS, viral RNA-binding proteins, viral factories, viral genetics

## Abstract

The function of the mammalian orthoreovirus (reovirus) σNS nonstructural protein is enigmatic. σNS is an RNA-binding protein that forms oligomers and enhances the stability of bound RNAs, but the mechanisms by which it contributes to reovirus replication are unknown. To determine the function of σNS-RNA binding in reovirus replication, we engineered σNS mutants deficient in RNA-binding capacity. We found that alanine substitutions of positively charged residues in a predicted RNA-binding domain decrease RNA-dependent oligomerization. To define steps in reovirus replication facilitated by the RNA-binding property of σNS, we established a complementation system in which wild-type or mutant forms of σNS could be tested for the capacity to overcome inhibition of σNS expression. Mutations in σNS that disrupt RNA binding also diminish viral replication and σNS distribution to viral factories. Moreover, viral mRNAs only incorporate into viral factories or factory-like structures (formed following expression of nonstructural protein μNS) when σNS is present and capable of binding RNA. Collectively, these findings indicate that σNS requires positively charged residues in a putative RNA-binding domain to recruit viral mRNAs to sites of viral replication and establish a function for σNS in reovirus replication.

## INTRODUCTION

Viral factories are intracellular structures formed during infection that promote production of progeny virions. These neoorganelles concentrate viral and host components to establish discrete intracellular environments that are optimal for viral genome replication, packaging, and often immune evasion or suppression (1). While it is known that factories require viral proteins and RNAs, mechanisms by which these viral components are recruited and concentrated are not well understood, especially for viruses that contain double-stranded (ds) RNA genomes.

Mammalian orthoreovirus (reovirus) has been implicated in the development of celiac disease (2) and is being investigated as an oncolytic therapeutic (3). Mature reovirus virions package 10 unique segments of dsRNA in two concentric shells, termed outer capsid and core (4). Following reovirus internalization into the endocytic compartment, the outer capsid is proteolytically removed, allowing penetration of the viral core into the cytoplasm (5). The core transcribes viral mRNAs that are translated by host ribosomes. Newly synthesized viral proteins reorganize the cytoplasm, establishing dynamic factories (6, 7) embedded in a matrix of membranes derived from the endoplasmic reticulum (6–8). Formation of these reovirus replication organelles requires two nonstructural proteins, μNS and σNS, which are expressed at early stages of infection (9). As infection progresses, additional viral replication components are concentrated in the factories, increasing their size (10). How these essential viral components are recruited to factories is not well defined.

The μNS protein forms the scaffold for reovirus factories (11). Expression of μNS in cells leads to the formation of dynamic globular structures that resemble liquid-liquid phase-separated condensates (7, 12). These structures do not require other reovirus proteins to form and appear similar in morphology to reovirus factories (9). As such, μNS puncta in uninfected cells are called factory-like structures (13). Protein components of reovirus cores are recruited to factory-like structures when coexpressed with μNS, suggesting that μNS concentrates reovirus proteins essential for viral assembly (14).

The σNS protein is a single-stranded (ss) RNA-binding protein that distributes diffusely in the cytoplasm when expressed alone but localizes to factory-like structures in the presence of μNS (9). Recruitment of σNS to μNS puncta requires the N-terminal regions of both proteins (15). Purified σNS interacts preferentially with ssRNAs and does not display sequence specificity (16). It is not known whether σNS binds ssRNA nonspecifically in infected cells. However, σNS can immunoprecipitate all 10 viral mRNAs from reovirus-infected cells and protects specific regions of viral mRNAs from degradation (17, 18). The N-terminal 38 residues of σNS are required for RNA binding (19), suggesting that this region forms an RNA-binding domain, although the mechanism by which σNS binds RNA and the function of its RNA-binding capacity in viral replication are unknown. Since this reovirus protein binds ssRNAs and localizes to viral factories, we hypothesized that it recruits viral mRNAs to these organelles.

In this study, we identified residues required for σNS binding to ssRNA by using targeted mutagenesis and found that these residues are required for efficient reovirus replication. Additionally, we discovered that viral mRNAs localize to viral factories and factory-like structures only when wild-type (WT) σNS is present. Collectively, our findings suggest that a function of the enigmatic σNS protein is to act in concert with μNS to recruit viral mRNAs to sites of viral replication.

## RESULTS

### Engineering σNS mutants deficient in RNA binding.

**FIG 1:**
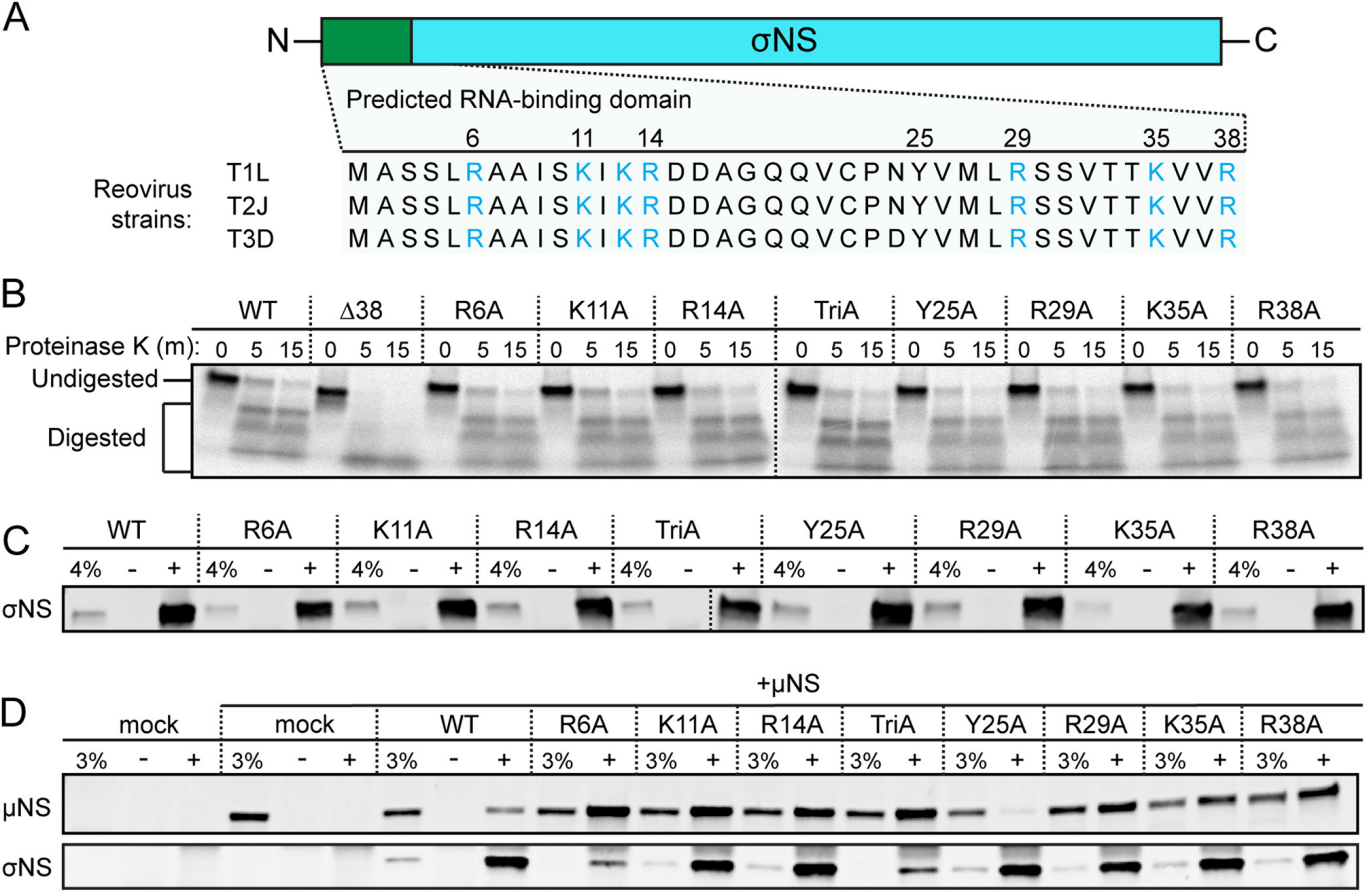
Mutations in σNS do not appear to alter protein folding. (A) Sequence alignment of the N-terminal 38 amino acids (green bar) of σNS encoded by mammalian reovirus strains T1L, T2J, and T3D. Positively charged residues are shown in blue. (B) ^35^S-labeled *in vitro*-expressed σNS was incubated with proteinase K for the times shown, resolved by SDS-PAGE, and visualized by autoradiography. HEK293T cells were transfected with σNS alone (C) or cotransfected with σNS and μNS (D) and incubated for 24 h. Total protein in cell lysates was immunoprecipitated using an IgG isotype antibody (−), conformation-specific σNS-specific monoclonal antibody 2A9 (C; +), or σNS-specific monoclonal antibody 3E10 (D; +), resolved by SDS-PAGE, and immunoblotted using antisera directed against σNS (C and D) or μNS (D). Percentages of total lysates in the immunoprecipitation reactions (4% [C] or 3% [D]) were used as loading controls.

To determine whether the RNA-binding capacity of σNS is required for reovirus replication, we engineered σNS mutants deficient in binding RNA. The first 38 residues in the σNS N terminus (Fig. 1A) are required for binding to RNA *in vitro* (19). These residues are conserved in available σNS sequences (20), and three of these residues (R6, R14, and R29) are conserved in σNS proteins of other *Orthoreovirus* species (see Fig. S1 in the supplemental material). Protein-RNA contacts occur by electrostatic or base-stacking interactions (21), and approximately 25% of the residues in this region of σNS are capable of mediating both types of interactions. We hypothesized that disrupting electrostatic or base-stacking interactions would prevent σNS from binding RNA. To test this hypothesis, we engineered seven alanine substitution mutations individually into a σNS expression plasmid, resulting in seven σNS mutants (R6A, K11A, R14A, Y25A, R29A, K35A, and R38A). We also engineered a triple mutant to disrupt a cluster of positively charged residues in the N terminus (K11A, K13A, and R14A; termed TriA) and a mutant lacking the N-terminal 38 residues (Δ38).

10.1128/mBio.01408-21.1 FIG S1 Sequence alignment of the N-terminal 38 amino acids (green bar) of σNS proteins encoded by *Orthoreovirus* species. Shown are σNS sequences from mammalian reovirus strains T1L, T2J, and T3D, avian reovirus (ARV), baboon reovirus (BRV), Nelson Bay orthoreovirus (NBV), and reptilian reovirus (RRV). Positively charged residues are shown in blue. Download FIG S1, PDF file, 0.1 MB. Copyright © 2021 Lee et al. 2021 Lee et al. https://creativecommons.org/licenses/by/4.0/ This content is distributed under the terms of the Creative Commons Attribution 4.0 International license.

To verify that the engineered σNS mutations do not disrupt protein folding, we characterized the mutants using three independent approaches. First, we conducted limited proteolysis of WT and mutant σNS proteins recovered from coupled *in vitro* transcription and translation reactions using rabbit reticulocyte lysates supplemented with ^35^S methionine. Following *in vitro* expression, WT and mutant proteins were digested with proteinase K, and digestion reactions were resolved by polyacrylamide gel electrophoresis (PAGE) (Fig. 1B). The only mutant that differed from WT σNS in digestion kinetics or resultant protein fragments was Δ38 σNS. Based on these results, we concluded that Δ38 σNS is not properly folded and excluded this mutant from subsequent analyses. Second, we used σNS conformation-specific monoclonal antibody 2A9 (10) to immunoprecipitate σNS expressed in HEK293T cells (Fig. 1C). While various levels of protein expression were apparent, all of the mutants were immunoprecipitated by this conformation-specific antibody, suggesting that the mutations do not disrupt an epitope in σNS recognized by this antibody. Third, we tested whether the mutant σNS proteins were capable of co-immunoprecipitation (co-IP) with a known σNS-binding partner, reovirus μNS protein. The two proteins were coexpressed in HEK293T cells, and co-IPs were conducted using monoclonal antibody 3E10, which also is directed against σNS (10) (Fig. 1D). Each of the mutants was capable of immunoprecipitating μNS. Surprisingly, replacing positively charged residues in σNS with alanine residues promoted more efficient immunoprecipitation of μNS. Collectively, the σNS alanine substitutions and the TriA σNS mutant yielded folding phenotypes comparable to that of WT σNS and were capable of interacting with a known σNS-binding partner, suggesting that the mutations do not substantially alter σNS structure.

**FIG 2:**
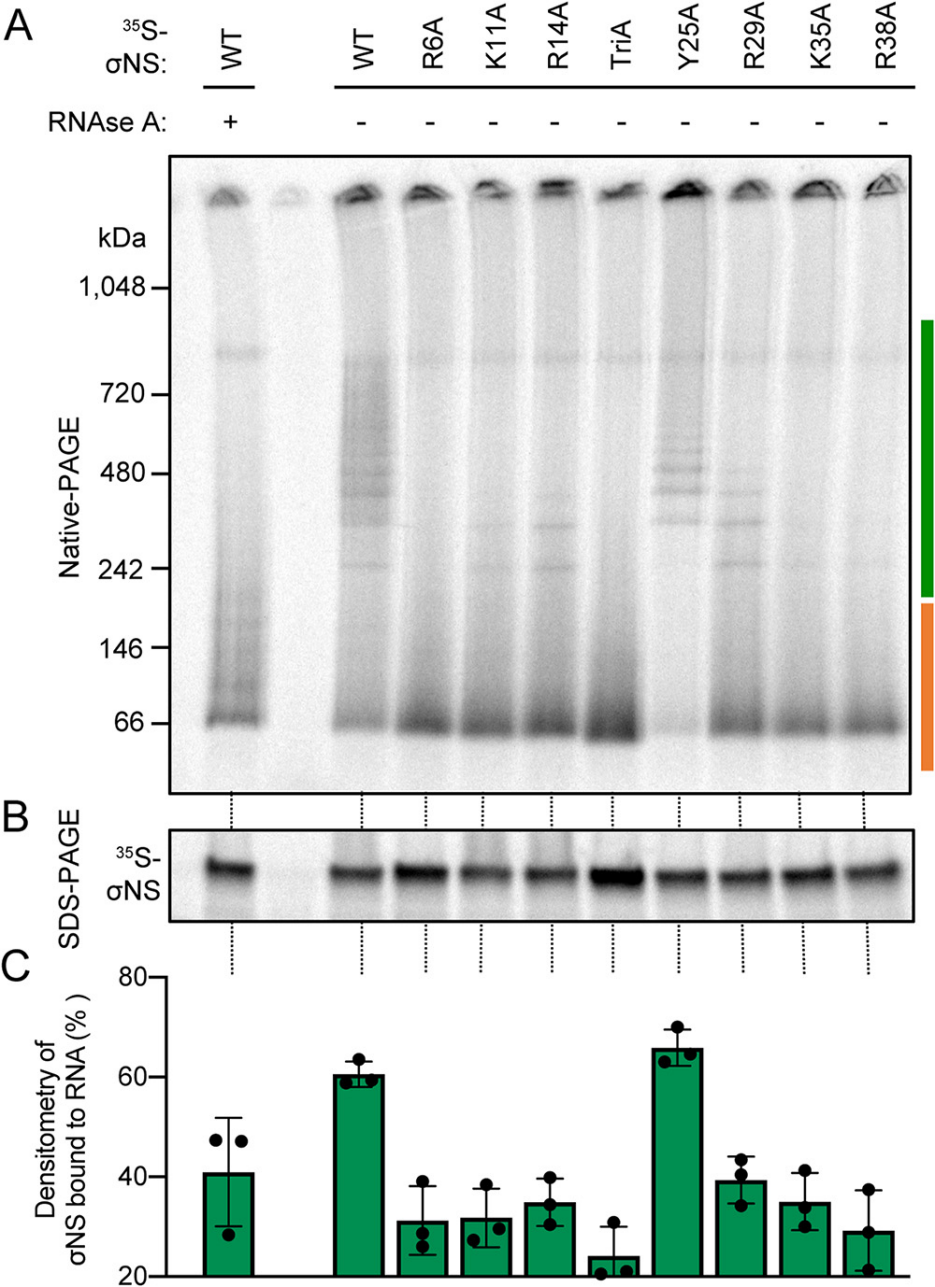
Alanine substitution of positively charged residues in a predicted RNA-binding domain of σNS alters RNA-dependent oligomerization. ^35^S-labeled σNS was expressed in rabbit reticulocyte lysates (RRLs) and incubated with or without RNase A. Samples were resolved by native PAGE to preserve oligomeric species (A) or SDS-PAGE to monitor protein expression (B) and visualized by autoradiography. The scale bar to the left of the native polyacrylamide gel (A) marks the kilodalton (kDa) ranges used for densitometric analysis of each σNS construct. The green scale bar marks σNS bound to RNA, whereas the orange scale bar marks unbound σNS. (C) The efficiency with which each σNS construct forms RNA-dependent oligomers was calculated by dividing the density of σNS bound to RNA (panel A, green scale bar) by the total density of σNS present in the gel (panel A, green and orange scale bar).

We next evaluated the RNA-binding capacity of the σNS mutants using an RNA-dependent electrophoretic mobility shift assay (EMSA). In these experiments, we employed a property of σNS to form oligomeric ladders with RNA when expressed *in vitro* (19). These ladders collapse following treatment with RNase A (Fig. 2A), indicating that RNA binding is required for ladder formation. WT and mutant σNS proteins were expressed in rabbit reticulocyte lysates in the presence of ^35^S methionine. Half of each protein sample was resolved using native PAGE to separate complexes of σNS and RNA, while the other half was electrophoresed using denaturing PAGE to compare protein levels (Fig. 2B). Based on the molecular weight of monomeric WT σNS (∼37 kDa), σNS appears to migrate in the presence of RNA as a hexamer and correspondingly larger species that vary by two monomers of σNS each. Treatment of the σNS-RNA complexes with RNase A yielded a band that migrates at approximately the 66-kDa-molecular-weight marker, which likely represents a dimer of σNS not bound to RNA. Mutant forms of σNS produced bands that migrated at comparable molecular weights to the dimer, but the intensities varied, inversely correlating with the intensities of the higher-molecular-weight bands. The percentage of RNA-dependent oligomers for each σNS protein was determined by dividing the total density of bands migrating between ∼200 and 1,000 kDa (Fig. 2A, green bar) by the sum of the densities of all bands observed in the gel (Fig. 2A, green and orange bars). Two mutants, R6A and TriA σNS, did not display any detectable RNA-dependent oligomerization (Fig. 2A). The RNA-binding capacity of the other charged-to-alanine mutants was less than that of WT or Y25A σNS (Fig. 2C). However, all σNS-RNA complexes were sensitive to RNase A treatment. Most mutants yielded dominant dimer bands following RNase A treatment, but the Y25A mutant did not. Instead, Y25A σNS appeared to aggregate at the top of the gel following RNase A treatment (see Fig. S2). These results suggest that positively charged residues in the σNS N terminus are required for RNA binding. However, predicted base-stacking interactions mediated by Y25 appear dispensable for this property, but without RNA, the Y25A mutant appeared more prone to aggregation.

10.1128/mBio.01408-21.2 FIG S2 RNase A treatment of σNS mutants disrupts RNA-dependent oligomerization. ^35^S-labeled σNS was expressed in RRLs and incubated with RNase A. Samples were resolved by native PAGE to preserve oligomeric species (A) or SDS-PAGE to monitor protein expression (B) and visualized by autoradiography. Download FIG S2, PDF file, 0.1 MB. Copyright © 2021 Lee et al. 2021 Lee et al. https://creativecommons.org/licenses/by/4.0/ This content is distributed under the terms of the Creative Commons Attribution 4.0 International license.

### Mutants of σNS incapable of RNA binding fail to complement σNS knockdown during infection.

To test whether σNS RNA-binding capacity contributes to reovirus replication, we first attempted to recover reoviruses encoding the R6A, K11A, or R29A mutations in σNS using reverse genetics. Plaque-forming mutant viruses were not recovered in three independent attempts, suggesting that residues required for RNA binding are also required for viral replication. To define the step in reovirus replication facilitated by the RNA-binding capacity of σNS, we established a complementation system in which WT or mutant forms of the proteins could be tested for the capacity to overcome inhibition of σNS expression. First, we evaluated the capacity of expressed WT or mutant σNS proteins to complement σNS knockdown in HEK293T cells constitutively expressing σNS-specific small interfering RNAs (σNS-siRNA cells) (19). As a control, we used HEK293T cells constitutively expressing siRNAs directed against green fluorescent protein (GFP-siRNA cells) (19). We transfected these cells with expression plasmids encoding GFP as a negative control, WT σNS, or σNS incorporating synonymous mutations in the siRNA recognition site (σNS mismatch [σNS-MM]). Transfected cells were incubated for 24 h, after which time, cells were adsorbed with reovirus at a low multiplicity of infection (MOI). We used a low MOI in these experiments to enable the constitutively expressed siRNAs to diminish the expression of virus-encoded σNS transcripts. As anticipated, at 24 h postadsorption, σNS protein levels (Fig. S3A) and reovirus replication (Fig. S3B) were unaffected by the GFP-restricting siRNA. However, σNS protein expression was not detected and reovirus replication was substantially impaired by the σNS-restricting siRNA, and neither σNS expression nor reovirus replication was complemented by GFP. σNS protein expression was greater following σNS-MM transfection relative to that following WT σNS transfection (Fig. S3A), demonstrating the susceptibility of WT σNS transcripts to siRNA-mediated knockdown in these cells. However, transfection of WT σNS into reovirus-infected σNS-siRNA cells allowed reovirus yields to reach levels comparable to those following transfection of σNS MM, indicating that increased levels of σNS allowed by the mismatch mutations in the siRNA target sequence do not lead to increased production of viral progeny (Fig. S3B). Both WT and MM σNS were capable of promoting reovirus replication in σNS-siRNA cells relative to that for complementation with GFP. These results indicate that overexpression of σNS prior to infection can rescue reovirus replication in cells expressing σNS-specific siRNAs and that rescue is independent of mismatch mutations in the σNS siRNA target sequence.

10.1128/mBio.01408-21.3 FIG S3 Overexpression of σNS complements siRNA knockdown of σNS during infection. Cells that constitutively express siRNAs directed against σNS or GFP were transfected with expression plasmids encoding GFP, WT σNS (σNS), or WT σNS with σNS-siRNA-resistant sequences (σNS MM) and incubated for 24 h. Cells were adsorbed with reovirus strain T3D at an MOI of 5 PFU/cell and incubated for 24 h. Cell lysates were collected for immunoblotting (A) and infectious virus quantification by plaque assay (B). (A) Immunoblot analysis of proteins expressed following complementation. Protein expression was evaluated using monoclonal antibodies specific for GFP or alpha-tubulin (α-tub) and guinea-pig sera specific for σNS. (B) Infectious virus quantification following complementation. Titer values that differ significantly from those obtained from cells expressing siRNAs against σNS complemented with GFP by one-way analysis of variance (ANOVA) with Dunnett’s multiple-comparison test are shown. ****, *P* < 0.0001. Download FIG S3, PDF file, 0.2 MB. Copyright © 2021 Lee et al. 2021 Lee et al. https://creativecommons.org/licenses/by/4.0/ This content is distributed under the terms of the Creative Commons Attribution 4.0 International license.

**FIG 3:**
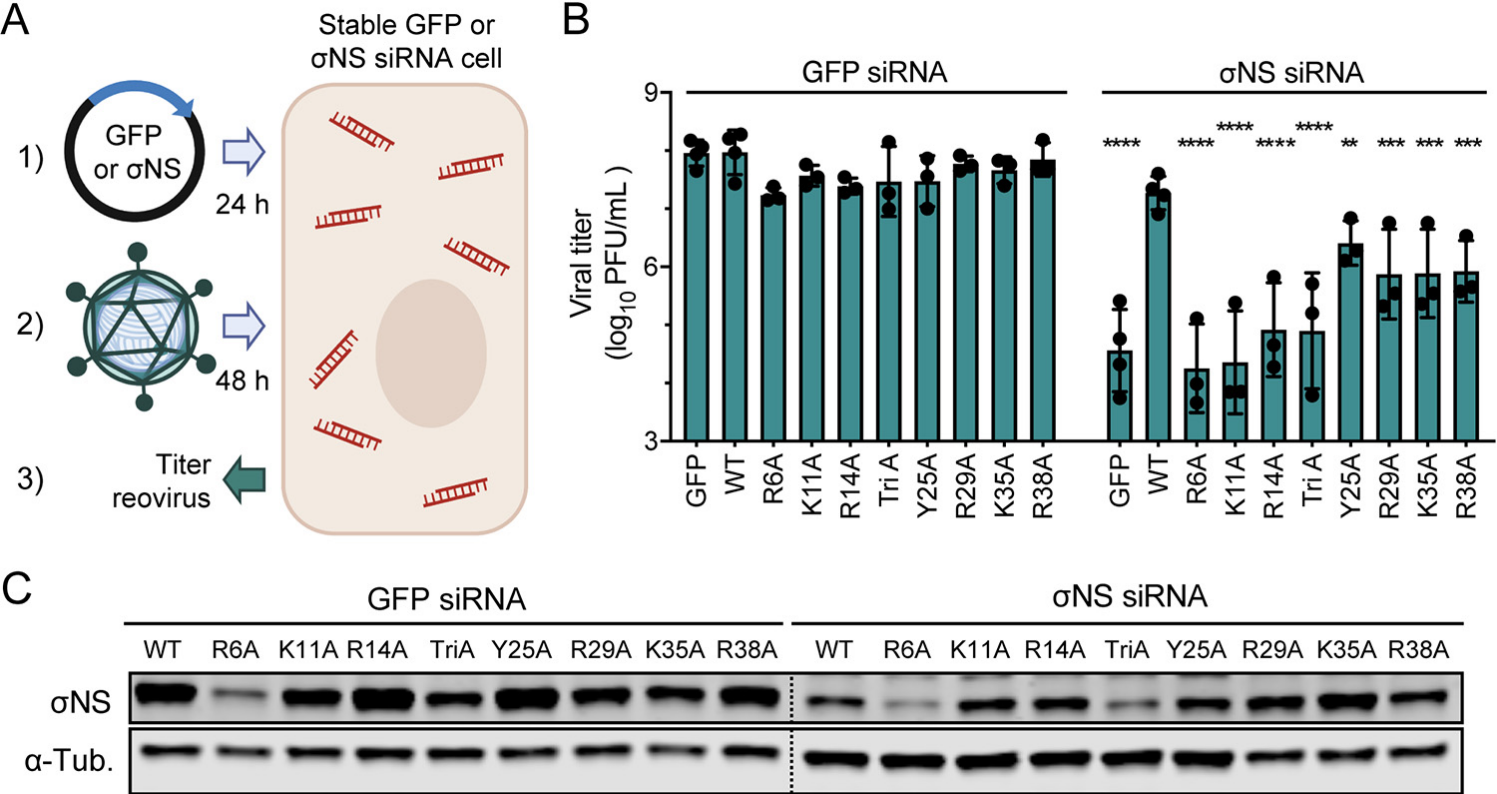
Mutants of σNS incapable of RNA binding fail to complement reovirus replication in cells expressing σNS-specific siRNAs. (A) Cells that constitutively express siRNAs directed against GFP or σNS were transfected with expression plasmids encoding GFP or the σNS constructs shown and incubated for 24 h. Cells were adsorbed with reovirus strain T3D at an MOI of 5 PFU/cell and incubated for 48 h. (B) Cell culture supernatants were collected for infectious virus quantification by plaque assay. Titer values that differ significantly from those obtained from cells expressing siRNAs against GFP complemented with GFP by one-way ANOVA with Dunnett’s multiple-comparison test are shown. **, *P* < 0.0021; ***, *P* < 0.0002; ****, *P* < 0.0001. (C) Cells that constitutively express siRNAs against GFP or σNS were transfected with expression plasmids encoding the σNS constructs shown and incubated for 24 h. Cell lysates were resolved by SDS-PAGE and immunoblotted using antisera directed against σNS and monoclonal antibodies specific for alpha-tubulin (α-Tub). Panel A was prepared using BioRender.

To determine whether the engineered σNS mutants can complement reovirus replication in cells expressing σNS-specific siRNAs, we transfected GFP-siRNA and σNS-siRNA cells with the σNS mutants, adsorbed with reovirus, and monitored viral yields by plaque assay (Fig. 3A). Following infection of GFP-siRNA cells, viral yields were only modestly altered by expression of WT or mutant σNS (Fig. 3B). In contrast, following infection of σNS-siRNA cells, viral yields were reduced to a maximum of 10,000-fold following expression of the σNS mutants incapable of binding RNA relative to expression of WT σNS (Fig. 3B). Additionally, Y25A σNS complemented reovirus replication more efficiently than the other mutants, albeit at lower levels than WT σNS. Transfection of GFP-siRNA and σNS-siRNA cells with σNS mutants resulted in variable levels of σNS protein after 24 h of incubation (Fig. 3C). R6A σNS and TriA σNS displayed the lowest levels of expression, which was surprising, as expression of these mutants was similar to that of WT σNS in rabbit reticulocyte lysates (Fig. 2B). However, levels of σNS present prior to infection did not correlate with the capacity of the mutants to complement σNS siRNA-mediated knockdown (Fig. 3B). K11A σNS, which was expressed at levels comparable to WT σNS, and R14A σNS, which was expressed at higher levels than WT σNS, were incapable of restoring viral replication. These results suggest that the capacity of σNS to support viral replication is not strictly contingent on levels of σNS expression but instead on properties of the protein that were altered following mutagenesis, likely, the capacity to bind RNA.

### σNS incorporation into reovirus factories is disrupted by mutations that alter RNA binding.

**FIG 4:**
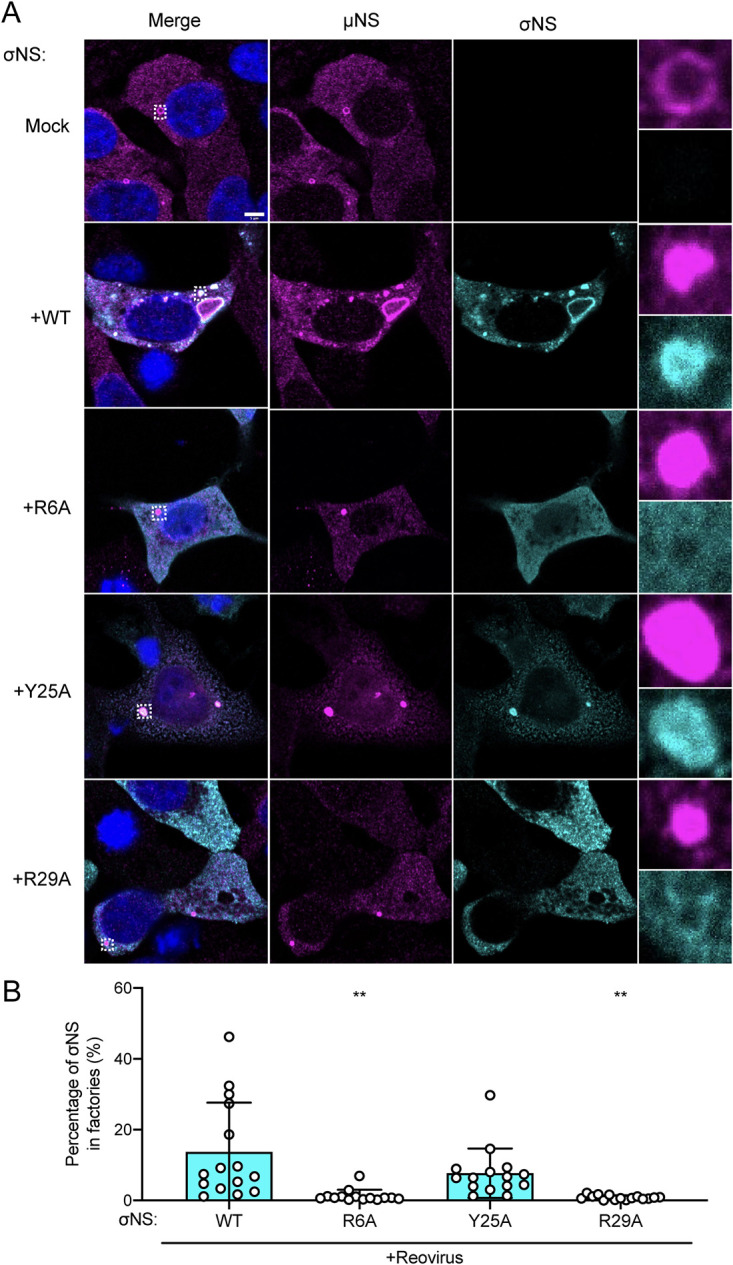
Mutations in σNS that compromise RNA binding disrupt incorporation of the protein into reovirus replication organelles. (A) Cells that constitutively express siRNAs directed against σNS were transfected with expression plasmids encoding the σNS constructs shown and incubated for 24 h. Cells were adsorbed with reovirus strain T3D at an MOI of 5 PFU/cell, incubated for 48 h, fixed, stained using σNS-specific monoclonal antibody 3E10 (cyan), μNS-specific antiserum (magenta), and DAPI (blue), and imaged using confocal microscopy. Regions selected for magnification are indicated by dotted white boxes. Bar, 5 μm. (B) The percentage of σNS immunofluorescence signal in reovirus factories was quantified by dividing the sum of σNS signal in reovirus factories by the sum of cytoplasmic σNS signal. Individual data points represent single cells. Percentage values that differ significantly from those obtained from WT σNS-transfected reovirus-infected cells by one-way analysis of variance (ANOVA) with Dunnett’s multiple-comparison test are shown. **, *P* < 0.0021.

In reovirus-infected cells, σNS preferentially localizes to viral factories (10). To determine whether σNS distribution in cells contributes to its function, we assessed the intracellular distribution of WT and mutant forms of σNS during infection. We selected three mutants (R6A, Y25A, and R29A) to represent our panel of σNS mutants in this and subsequent experiments. The R6A and R29A mutants displayed little to no RNA-dependent oligomerization and failed to complement σNS knockdown in infected σNS-siRNA cells (Fig. 3B). The Y25A mutant displayed RNA-dependent oligomerization comparable to that of WT σNS and complemented σNS knockdown in infected σNS-siRNA cells more efficiently than the other σNS mutants. To evaluate the distribution of mutant σNS during infection, we transfected σNS-siRNA cells with WT or mutant forms of σNS, infected them with reovirus, stained them with antibodies specific for σNS and μNS, and imaged the cells using confocal immunofluorescence microscopy (Fig. 4A). Viral factory structures were demarcated by intense μNS staining. Factories formed under all conditions tested and retained a globular morphology, which is characteristic of the type 3 Dearing (T3D) strain of reovirus used in these experiments (22). However, factories formed in the absence of σNS expression or in the presence of the R6A or R29A σNS mutants were smaller than those formed in the presence of WT or Y25A σNS. Immunofluorescence signals for WT and Y25A σNS were more frequently detected in viral factories, while those produced by the R6A and R29A σNS mutants were more frequently detected outside viral factories (Fig. 4B). Collectively, these observations suggest that mutations impairing RNA binding limit viral factory maturation and alter σNS distribution to viral factories.

To confirm that the preferential distribution of σNS to the periphery of larger viral factories was not solely due to poor penetration of σNS-specific antibodies into factories of fixed and permeabilized cells, we processed reovirus-infected cells for Tokuyasu cryosections, stained σNS with gold-labeled σNS-specific antibodies, and imaged the cells using transmission electron microscopy (Fig. S4). Small puncta containing σNS were observed throughout the cytoplasm in the majority of infected cells. These small puncta were not coated at the periphery with σNS, and instead, σNS was distributed diffusely in these structures (Fig. S4A and B). However, in larger electron-dense factories, σNS was concentrated at the factory periphery (Fig. S4C and D), consistent with previous results (23). These observations suggest that σNS distributes to the factory periphery as these structures enlarge.

10.1128/mBio.01408-21.4 FIG S4 Immunogold labeling of σNS proteins in Tokuyasu cryosections of reovirus-infected cells. Cells were adsorbed with reovirus strain T1L M1-P208S at an MOI of 1 PFU/cell, incubated for 14 h, frozen in liquid nitrogen, and sectioned at −120°C. Thawed cryosections were processed for immunogold labeling using σNS-specific monoclonal antibody 2F5, followed by a secondary antibody bound to 10-nm colloidal gold spheres. Cryosections were imaged using transmission electron microscopy. (A and B) Representative images of small, punctate viral factories. (C and D) Representative images of larger mature factories. Nucleus (N), endoplasmic reticulum (ER), and mitochondria (mi) are labeled when visible surrounding a viral factory (*). Bars, 50 nm (A and B), 200 nm (C and D). Download FIG S4, PDF file, 0.2 MB. Copyright © 2021 Lee et al. 2021 Lee et al. https://creativecommons.org/licenses/by/4.0/ This content is distributed under the terms of the Creative Commons Attribution 4.0 International license.

**FIG 5:**
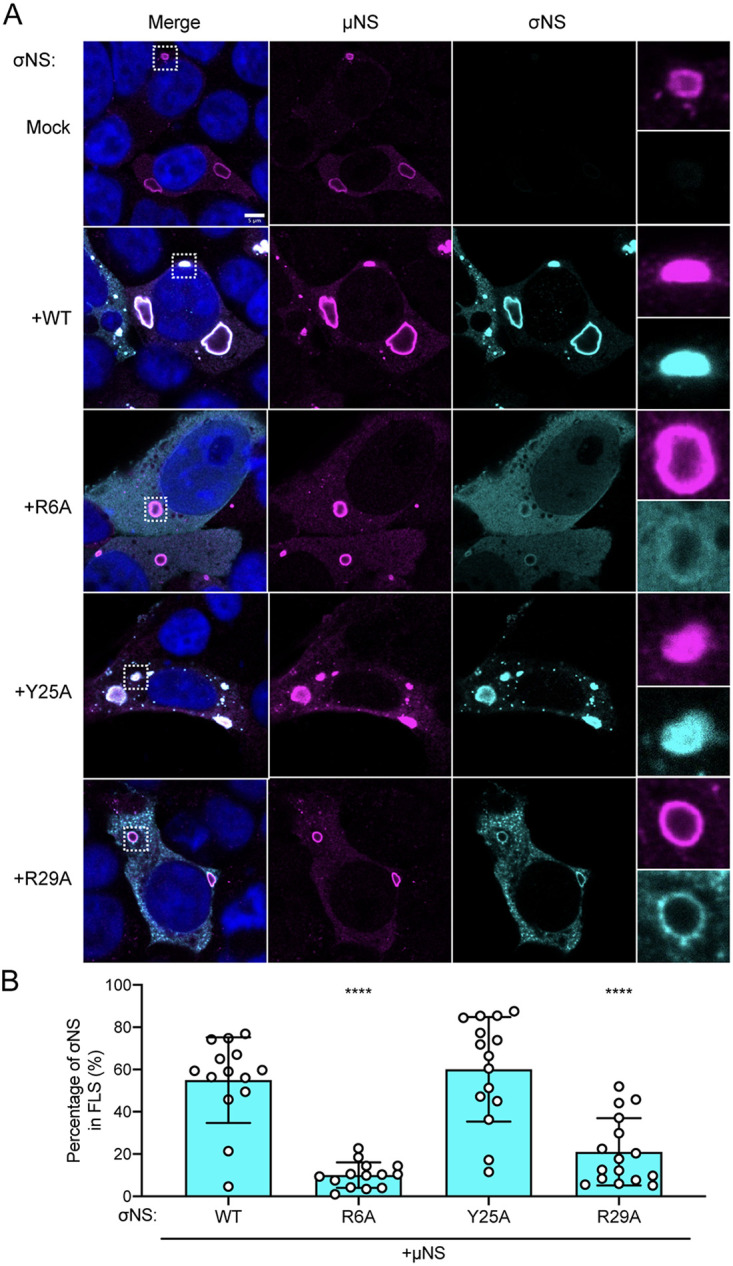
Mutations in σNS that compromise RNA binding disrupt incorporation of the protein into factory-like structures. (A) Cells were transfected with expression plasmids encoding the σNS constructs shown along with μNS and incubated for 24 h. Cells were fixed, stained using σNS-specific monoclonal antibody 3E10 (cyan), μNS-specific antiserum (magenta), and DAPI (blue), and imaged using confocal microscopy. Regions selected for magnification are indicated by dotted white boxes. Bar, 5 μm. (B) The percentage of σNS immunofluorescence signal in reovirus factory-like structures was quantified by dividing the sum of σNS signal in reovirus factory-like structures by the sum of cytoplasmic σNS signal. Individual data points represent single cells. Percentage values that differ significantly from those obtained from WT σNS and μNS cotransfected cells by one-way analysis of variance (ANOVA) with Dunnett’s multiple-comparison test are shown. ****, *P* < 0.0001.

To determine whether incorporation of σNS into reovirus factories depends on viral replication, we used a simplified factory-like structure model system (9, 11). We transfected HEK293T cells with μNS and WT or mutant forms of σNS and processed the cells for confocal immunofluorescence microscopy to visualize σNS and μNS (Fig. 5A). WT and Y25A σNS were efficiently incorporated into factory-like structures (Fig. 5B). The morphology and size of these structures resembled those of viral factories observed during reovirus infection (Fig. 5B). In contrast, the R6A and R29A σNS mutants were poorly incorporated into factory-like structures (Fig. 5B) and recapitulated phenotypes observed during complementation of reovirus-infected cells (Fig. 4B). Based on data presented thus far, we conclude that RNA promotes oligomerization of σNS, which could enable a greater number of σNS molecules to incorporate into factory structures, overcoming saturation limits of μNS binding.

### Mutations in σNS that alter RNA binding diminish mRNA incorporation in reovirus factories.

**FIG 6:**
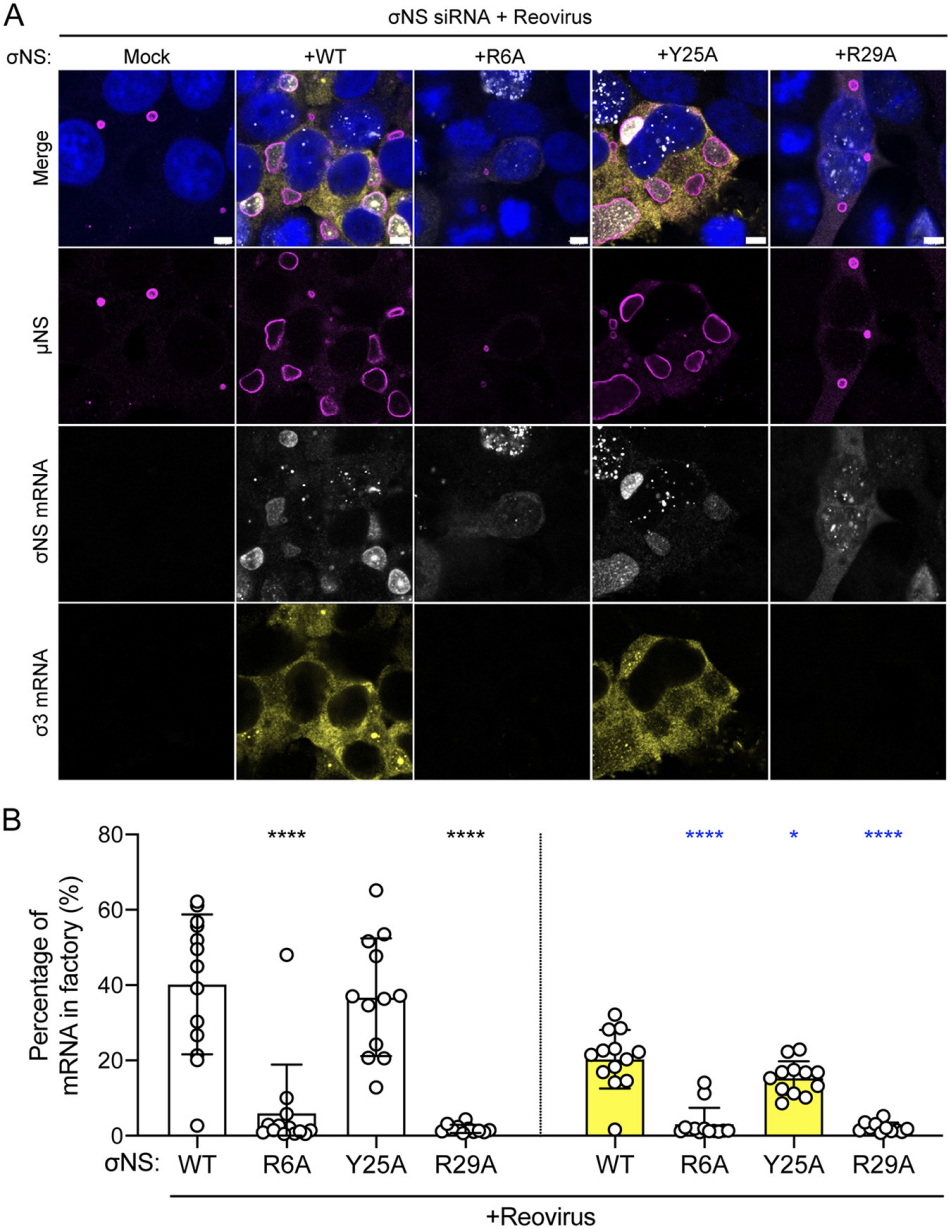
Reovirus transcripts are present in viral factories only when σNS is capable of binding RNA. Cells that constitutively express siRNAs directed against σNS were transfected with expression plasmids encoding the σNS constructs shown and incubated for 24 h. Cells were adsorbed with reovirus strain T3D at an MOI of 5 PFU/cell, incubated for 48 h, fixed, stained using RNA FISH probes specific for σNS mRNA (white) or σ3 mRNA (yellow), μNS-specific antiserum (magenta), and DAPI (blue), and imaged using confocal microscopy. Bar, 4.8 μm. The percentage of cytoplasmic σNS-mRNA (B, white bars) and σ3-mRNA (B, yellow bars) FISH signals in reovirus factories was quantified by dividing the sum of the FISH signal in reovirus factories by the sum of the cytoplasmic FISH signal. Individual data points represent single cells. Percentage values that differ significantly from those obtained from WT σNS-transfected reovirus-infected cells by one-way analysis of variance (ANOVA) with Dunnett’s multiple-comparison test are shown. *, *P* < 0.0332; ****, *P* < 0.0001.

Since the R6A and R29A σNS mutants are altered in RNA binding and incorporation into viral factories, we hypothesized that viral mRNAs also are mislocalized in infected σNS-siRNA cells transfected with these mutants. To test this hypothesis, we transfected σNS-siRNA cells with WT or mutant forms of σNS, infected them with reovirus, and processed the cells 48 h postadsorption for fluorescence *in situ* hybridization (FISH) coupled with immunofluorescence detection of μNS to visualize reovirus factories (Fig. 6A). FISH probes were designed to specifically detect either the reovirus σNS-encoding mRNA (σNS mRNA) or the reovirus σ3-encoding mRNA (σ3 mRNA), which encodes outer-capsid protein σ3. Following infection of σNS-siRNA cells by reovirus, viral mRNAs were not detected in viral factories. Expression of WT and Y25A σNS prior to infection led to the formation of larger viral factories than in untransfected cells, and importantly, both σNS and σ3 mRNAs were detected in cells. Interestingly, σ3 mRNAs were not concentrated in factories to the same extent as σNS mRNAs, but both σNS and σ3 mRNAs were observed to colocalize in discrete high-intensity puncta in viral factories, suggesting a suborganization in the factory structures. While expression of WT and Y25A σNS promoted conditions to allow detection of viral mRNAs in factories, expression of the R6A and R29A σNS mutants did not. R6A and R29A σNS mRNAs were detected in cells containing small factories, but these mRNAs were not observed in the factory structures. These results suggest that viral mRNAs are not efficiently produced or do not distribute to factories when σNS is incapable of interacting with RNA.

We were surprised that σ3 mRNAs were not concentrated in functioning factories to the same extent as σNS mRNAs. We hypothesized that differences in σNS and σ3 mRNA localization could be dependent on the time point at which we fixed cells for imaging. To test this hypothesis, we infected HEK293T cells with reovirus, and processed the cells at 9, 24, and 48 h postadsorption for FISH coupled with immunofluorescence detection of μNS to visualize reovirus factories (Fig. S5). Both σ3 and σNS mRNAs were concentrated in factories at 9 and 24 h postadsorption. However, at 48 h postadsorption, σ3 mRNAs distributed less prevalently to factories, while σNS mRNAs maintained a predominantly factory distribution. These results suggest that as viral factories mature, some viral mRNAs distribute to different intracellular sites.

10.1128/mBio.01408-21.5 FIG S5 Reovirus transcripts differentially localize in viral factories at late timepoints postadsorption. HEK293T cells were adsorbed with reovirus strain T3D at an MOI of 20 PFU/cell, incubated for 9, 24, and 48 h, fixed, stained using RNA FISH probes specific for σNS mRNA (white) or σ3 mRNA (yellow), μNS-specific antiserum (magenta), and DAPI (blue), and imaged using confocal microscopy. Bar, 5 μm. The percentage of cytoplasmic σNS-mRNA (B, white bars) and σ3-mRNA (B, yellow bars) FISH signals in reovirus factories was quantified by dividing the sum of the FISH signal in reovirus factories by the sum of the cytoplasmic FISH signal. Individual data points represent single cells. Percentage values that differ significantly from those obtained from those at 9 h postadsorption by one-way analysis of variance (ANOVA) with Dunnett’s multiple-comparison test are shown. *, *P* < 0.0332; ***, *P* < 0.0002; ****, *P* < 0.0001. Download FIG S5, PDF file, 0.2 MB. Copyright © 2021 Lee et al. 2021 Lee et al. https://creativecommons.org/licenses/by/4.0/ This content is distributed under the terms of the Creative Commons Attribution 4.0 International license.

### WT σNS and μNS are sufficient to recruit viral mRNA to factory-like structures.

**FIG 7:**
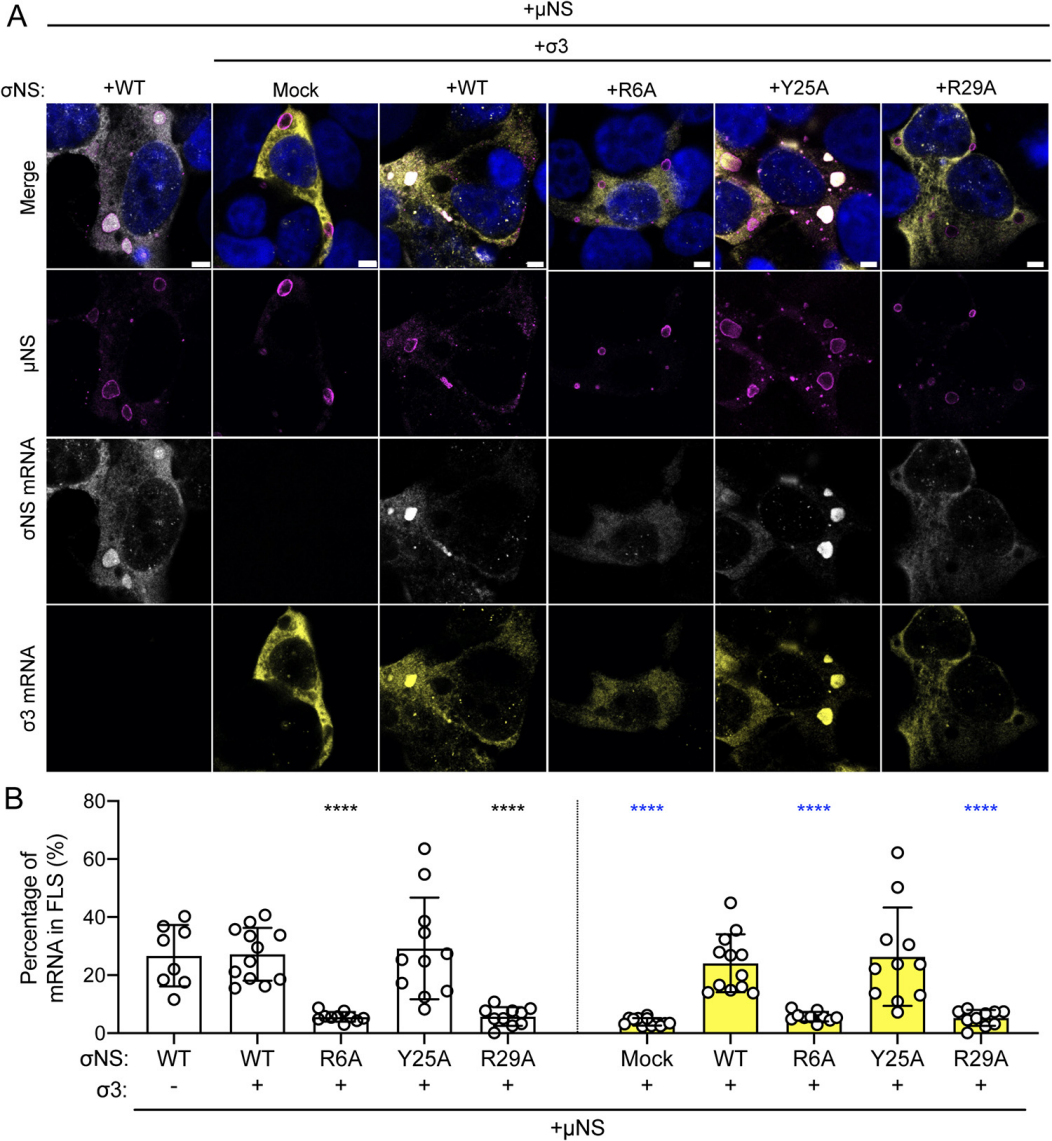
Reovirus mRNAs are recruited to factory-like structures by σNS. Cells were transfected with expression plasmids encoding μNS, σ3, and the σNS constructs shown and incubated for 24 h. Cells were fixed, stained using RNA FISH probes specific for σNS mRNA (white) or σ3 mRNA (yellow), μNS-specific antiserum (magenta), and DAPI (blue), and imaged using confocal microscopy. Bar, 4.8 μm. The percentage of cytoplasmic σNS-mRNA (B, white bars) and σ3-mRNA (B, yellow bars) FISH signals in factory-like structures was quantified by dividing the sum of the FISH signal in factory-like structures by the sum of the cytoplasmic FISH signal. Individual data points represent single cells. Percentage values that differ significantly from those obtained from cells cotransfected with WT σNS, σ3, and μNS by one-way analysis of variance (ANOVA) with Dunnett’s multiple-comparison test are shown. ****, *P* < 0.0001.

The lack of viral mRNAs in factories formed in the presence of mutant σNS could be independent of viral mRNA incorporation into viral factories and instead due to impaired viral replication, leading to reduced secondary rounds of viral transcription. To uncouple viral mRNA distribution from viral replication, we transfected HEK293T cells with different combinations of expression plasmids encoding μNS, WT or mutant forms of σNS, and σ3, fixed and stained them for FISH, and imaged the cells using confocal immunofluorescence microscopy (Fig. 7A). The σ3 protein binds double-stranded RNA but not single-stranded RNA (24) and, thus, would not be expected to retain viral mRNA in factory-like structures. Concordantly, expression of μNS and σ3 was insufficient to promote incorporation of σ3 mRNAs into factory-like structures (Fig. 7B, yellow). However, expression of μNS and σ3 along with WT or Y25A σNS led to concentration of σ3 mRNAs (Fig. 7B, yellow) as well as σNS mRNAs in these structures (Fig. 7B, white). Neither σNS nor σ3 mRNAs concentrated in factory-like structures following expression of the R6A or R29A σNS mutants with μNS and σ3. Instead, viral mRNAs appeared to be excluded from the interior of factory-like structures in the presence of these mutant σNS proteins. Since the plasmids are transcribed in the nucleus, these data suggest that σNS functions to recruit viral mRNAs into cytoplasmic factory-like structures. Collectively, these results suggest that σNS is required to recruit viral mRNAs to reovirus factories.

## DISCUSSION

**FIG 8:**
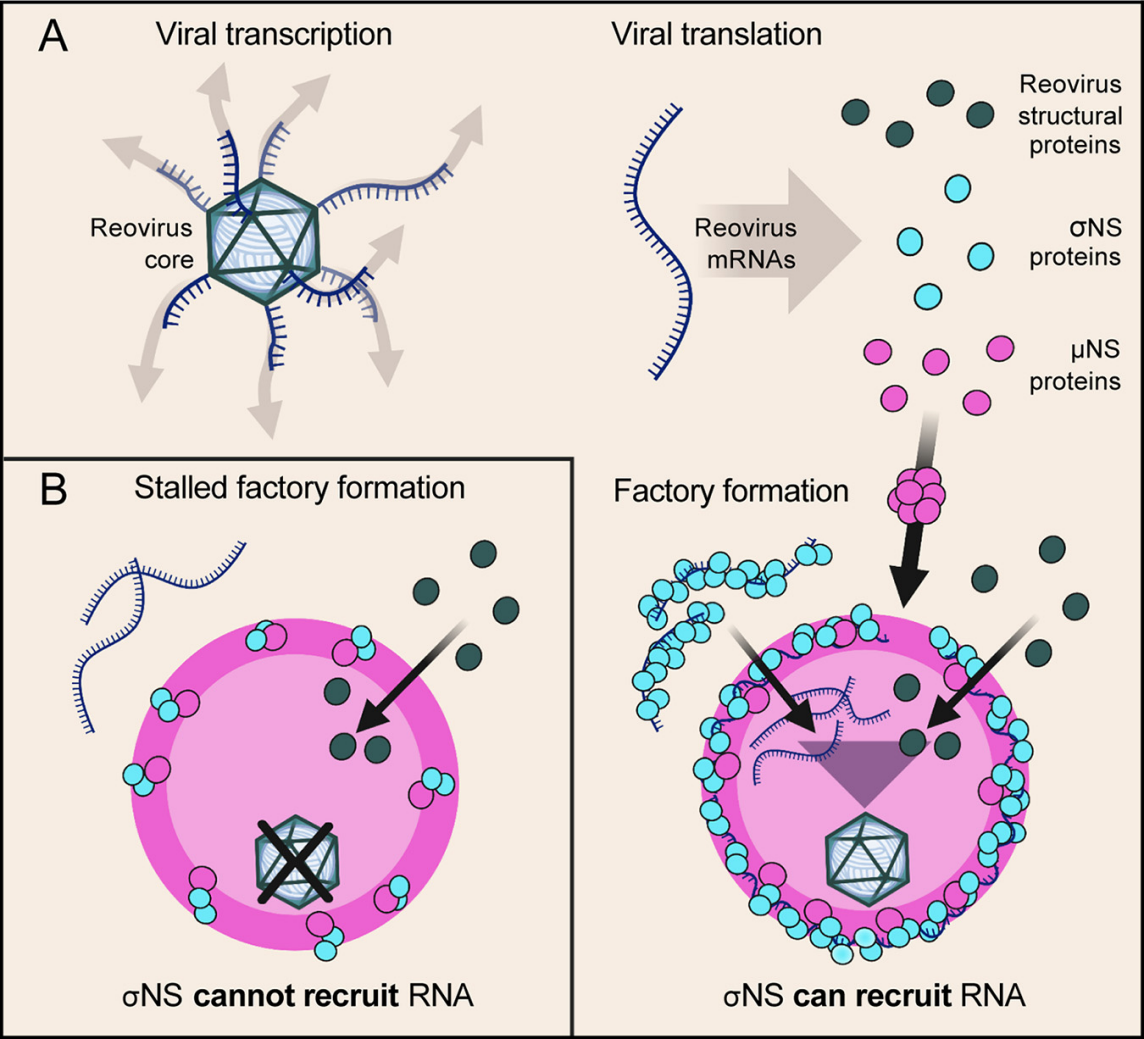
Model of σNS mRNA recruitment for reovirus factory formation. (A) Following viral attachment and internalization, reovirus cores enter the cytoplasm and transcribe viral mRNAs that are translated to yield structural and nonstructural (NS) proteins. Factory scaffolds are formed by μNS (large magenta sphere), which recruits reovirus structural proteins. Viral mRNAs in the cytoplasm are bound by σNS and delivered to factory scaffolds. Viral components that form nascent viral cores are concentrated in factories, promoting viral packaging and core maturation for secondary rounds of transcription and translation. (B) Mutations in σNS that compromise RNA-binding capacity also impede recruitment of mRNAs to factories and stall factory enlargement, as nascent cores do not form and are thus incapable of amplifying viral replication. This figure was prepared using BioRender.

In this study, we discovered a function for σNS in reovirus factory formation. We found that σNS requires electrostatic interactions to bind RNA and that RNA is not required to facilitate σNS-μNS interactions. We also observed that impeding σNS-RNA binding disrupts viral mRNA incorporation into viral factory scaffolds. Reovirus factories that form in the presence of σNS mutants incapable of binding RNA do not produce progeny viral particles. A model of σNS recruiting viral mRNAs for reovirus factory formation is shown in Fig. 8.

The manner in which RNA-binding proteins interact with RNA can influence the biological function of the resulting ribonucleoprotein complexes. Our data suggest that electrostatic interactions are required for reovirus σNS to bind RNA (Fig. 2). These interactions could occur between an RNA base or the RNA phosphate backbone and positively charged residues in the N-terminal region of σNS. Similarly, avian reovirus σNS also requires positively charged residues in its N terminus (R6 and R11) to bind ssRNAs *in vitro* (25). Avian reovirus σNS additionally displays RNA chaperone activity *in vitro* (26), suggesting another potential function for σNS is to fold viral mRNAs. Certain mRNA structures could enhance RNA-RNA interactions between different viral mRNA segments, allowing precise packaging of 10 unique viral mRNAs into progeny viral particles (4). Rotavirus NSP2, which has been hypothesized to be a functional homolog of σNS (26), also binds RNA using electrostatic interactions and, analogous to avian reovirus σNS, chaperones rotavirus mRNAs (25). NSP2 regulates RNA-binding using residues that electrostatically repulse RNA (27). While we have identified residues required for σNS to bind RNA, questions about the regulation and specificity of RNA binding remain. σNS may displace RNA similarly to NSP2 or by some type of posttranslational modification (28). Aside from cellular contexts, σNS does not preferentially bind viral mRNAs (16). However, in the context of viral infection, we hypothesize that σNS preferentially binds and concentrates viral mRNAs in factories. Concentrating host mRNAs in viral factories may compromise viral packaging, as viral mRNAs would have to compete with cellular mRNAs for RNA-RNA interactions. In support of this idea, RNA-binding specificity of other RNA-binding proteins differs *in vitro* and in cells (29). Future studies of σNS-RNA binding could help fill these knowledge gaps and explain how σNS controls the selective uptake of viral mRNAs into reovirus factories. Such a mechanism of control could be exploited to modulate the types of RNAs recruited into factory-like structures (30).

The charge-to-alanine mutations in the N terminus of σNS engineered in this study do not impair interactions of σNS with μNS as detected by co-immunoprecipitation (Fig. 1D). However, mutant forms of σNS incapable of RNA binding are not recruited to factories (Fig. 4A) or factory-like structures nucleated by μNS (Fig. 5A). There are three possible explanations for the inconsistency of our co-immunoprecipitation and colocalization results. First, since the same general N-terminal region of σNS (residues 1 to 11) is required for binding to RNA and μNS (13, 31), mutations that disrupt RNA binding may allow enhanced accessibility of that region to interact with μNS. Second, σNS could bind μNS proteins that have not integrated into the factory scaffolds. Third, σNS could bind μNS more avidly during cell lysis. The process of cell lysis likely disrupts the stability of factories and factory-like structures, which would allow increased access of σNS to μNS compared to that expected in viral factories.

Reovirus σNS is required for a step or steps in viral replication at or prior to dsRNA synthesis by the viral polymerase (19). Based on previous results, σNS interactions with viral mRNAs could enhance the stability of mRNAs bound at early stages of infection (19). However, based on our findings, we think that σNS is also required for the formation of functional viral factories, which precedes dsRNA synthesis (32). The morphology of viral factories does not change dramatically in the absence of σNS, but factories are notably smaller when σNS is absent (19) (Fig. 4). During viral factory morphogenesis, σNS likely alters factory scaffold properties to allow RNA incorporation. Viral mRNAs are thought to be packaged into nascent core particles in viral factories during assembly of reovirus progeny. σNS mutants incapable of binding RNA retain the capacity to be recruited to factory structures, albeit to a lesser degree. However, σNS distribution to factories is insufficient for its function in reovirus replication. σNS additionally requires RNA-binding capacity, which mediates incorporation of viral mRNAs into factories. The accumulation of viral mRNAs (recruited by σNS) and viral structural proteins (recruited by μNS) within factories establishes an environment replete with viral components. Progeny virions then can form and amplify viral replication to yield much larger factories.

While our findings enhance an understanding of σNS function during early steps in reovirus replication, questions remain about other potential functions of this protein. In addition to a potential role as an RNA chaperone, it is possible that σNS enhances interactions between the viral polymerase and mRNAs, as observed for other viral RNA-binding proteins (33–37). Any of these observed or potential functions require that σNS dissociate from viral mRNAs, as σNS is not contained in mature viral particles (38). The mechanism underlying mRNA release from σNS is not known, and it is not apparent at precisely what site in the cell such dissociation would occur. As factories enlarge, σNS concentrates at the factory periphery (23) (see Fig. S4 in the supplemental material), suggesting that σNS dissociates from RNA at that site. However, it also is possible that σNS dissociates from RNAs in the factory center, as the protein is detectable throughout factories, albeit in more limited quantities, especially in larger factories.

Formation of functional reovirus factories also requires cellular factors, many of which are unknown. Therefore, it is possible that σNS modifies host components in some way to promote factory formation and viral replication. Expression of σNS in the absence of other viral proteins induces endoplasmic reticulum (ER) tubulation (39). This morphological change is hypothesized to culminate in the formation of the ER fragments embedded in reovirus factories during infection. σNS could facilitate the incorporation of ER fragments into factories by binding ER-resident RNAs or proteins or engaging ER lipids. While the function of the ER fragments within factories has not been established, we think that the membranes provide a physical matrix to allow viral packaging, as observed for many RNA viruses (40). Proteins essential for the integrated stress response also are implicated in reovirus replication (41–43). In stressed cells, σNS recruits G3BP1 and other stress granule proteins to factory-like structures (44). The recruitment of stress granule proteins to factories depends on RNA and could lead to recruitment of the translational machinery, usually found within stress granules, to viral factories (23). These activities could occur concomitantly with viral mRNA recruitment by σNS.

Membrane enclosure is the most broadly known mechanism for organelles to compartmentalize intracellular components required for efficient molecular interactions and functions. However, organelles can form using a process of liquid-liquid phase separation (45). Liquid-liquid phase separation leads to the development of dynamic organelles, called condensates, that are stabilized by multivalent interactions between proteins, nucleic acids, or both (45). These organelles can separate from the intracellular environment, selectivity package discrete constituents, and allow biochemical activities, such as viral genome replication and capsid assembly, to be coordinated with high efficiency (46). Many RNA viruses use this mechanism to form viral factories (47–51). While development of many types of liquid-liquid phase-separated condensates of both viral and cellular origin depends on RNA, the formation of reovirus factories may differ (52). Since reovirus mRNAs are excluded from the factory-like structures formed solely by μNS, it is likely that μNS does not require RNA to phase separate. In this way, reovirus factory-like structures resemble other condensates that do not require RNA to mediate multivalent interactions (53, 54). For example, the formation of measles virus factories also does not require RNA, and a viral RNA-binding protein, N protein, can recruit RNA to factory-like structures to efficiently form RNP complexes within (50). Future studies will identify the minimal constituents and conditions required to form reovirus factory condensates and define the biophysical changes that occur when other components are added.

Experiments reported here indicate that σNS incorporates viral mRNAs into reovirus factory scaffolds that naturally exclude viral mRNAs. Our work begins to uncover how a dsRNA virus factory controls the selectivity of its composition. The next steps include defining the specificity of σNS interactions with RNA and identifying additional molecular interactants that enable σNS to promote viral genome replication and packaging. These studies are anticipated to illuminate new targets to impede dsRNA virus replication, which may have broad utility.

## MATERIALS AND METHODS

### Cells, viruses, and plasmids.

HEK293T cells and HEK293T cells expressing a GFP-specific siRNA (GFP-siRNA cells) (19) or an S3-specific siRNA (σNS-siRNA cells) (19) were maintained in Dulbecco’s modified Eagle’s medium (DMEM) supplemented to contain 5% fetal bovine serum (FBS) and 2 mM l-glutamine (Life Technologies). Culture medium for the siRNA-expressing cells was additionally supplemented to contain 5 μg/ml of puromycin (InvivoGen). L929 (L) cells adapted for growth in spinner cultures were maintained in Joklik’s minimal essential medium (JMEM) supplemented to contain 5% FBS, 1% l-glutamine, 50 U/ml of penicillin, 50 μg/ml of streptomycin (Life Technologies), and 0.25 μg/ml amphotericin B (Sigma). HeLa CCL2 cells were grown in DMEM supplemented to contain 10% FBS, 1% sodium pyruvate (Gibco), 1% MEM nonessential amino acids (Sigma-Aldrich), 1% l-glutamine, 50 U/ml of penicillin, 50 μg/ml of streptomycin, and 0.25 μg/ml amphotericin B.

WT reovirus strain T3D was recovered using reverse genetics (55). Site-directed mutagenesis of the reverse genetics plasmid encoding σNS was used to engineer σNS with R6A, K11A, and R29A mutations. Viruses encoding these mutations were not recovered using reverse genetics. Primers used for mutagenesis are listed in Table S1 in the supplemental material. Reovirus strain T1L M1-P208S (56) was recovered using reverse genetics (55). Reovirus T1L M1-P208S contains a point mutation in the M1 gene that causes viral factories to have a globular morphology similar to the morphology of factories formed by reovirus T3D (56). Viruses were amplified in L cells and purified by cesium chloride gradient centrifugation as described previously (57). Viral titers were determined by plaque assay using L cells (58).

10.1128/mBio.01408-21.6 TABLE S1 Primers used to clone a μNS expression plasmid and mutant forms of σNS. Download Table S1, PDF file, 0.1 MB. Copyright © 2021 Lee et al. 2021 Lee et al. https://creativecommons.org/licenses/by/4.0/ This content is distributed under the terms of the Creative Commons Attribution 4.0 International license.

Reovirus T3D σ3 (59), WT σNS (19), Δ38 σNS (19), and GFP (59) expression plasmids have been described elsewhere. T3D μNS expression plasmid was engineered by amplification of the T3D M3 open reading frame to contain 5′ KpnI and 3′ NotI restriction sites using reverse-genetics plasmid pT7-M3T3D (60) and T3D M3 5′-KpnI-NotI-3′ primers listed in Table S1. The amplified DNA was digested with NotI-HF and KpnI-HF (New England BioLabs [NEB]) and purified from agarose gel fragments following electrophoresis. The purified PCR product was ligated into pcDNA3.1+ vectors between the NotI-HF and KpnI-HF restriction sites. Site-directed mutagenesis was used to engineer σNS expression plasmids encoding R6A, K11A, R14A, TriA, Y25A, R29A, K35A, and R38A with primers listed in Table S1. Fidelity of cloning and mutagenesis was confirmed using Sanger sequencing (Genewiz) and Genewiz primers T7 and BGHR.

### Expression, proteolysis, and RNA-dependent oligomerization assays.

Reovirus σNS proteins were expressed from plasmids *in vitro* using the TNT T7 polymerase-coupled rabbit-reticulocyte lysate system (Promega, L4610) (19). Reactions were supplemented with [^35^S]methionine (Perkin Elmer, NEG709A500UC), incubated at 30°C for 1.5 h, and terminated with a 4-fold dilution in stop buffer (20 mM HEPES-KOH [pH 7.4], 100 mM potassium acetate, 5 mM magnesium acetate, 5 mM EDTA, 2 mM methionine, and freshly supplemented to contain 1 mM dithiothreitol [DTT] and 2 mM puromycin). Terminated reactions were used for proteolysis and RNA-dependent oligomerization assays.

Proteolysis assays were conducted by incubating translation reactions with 1 μg/ml proteinase K (Sigma) at 37°C for 0, 5, 10, or 15 min. Samples were prepared for sodium dodecyl sulfate-polyacrylamide gel electrophoresis (SDS-PAGE).

RNA-dependent oligomerization assays were conducted by incubating translation reactions with 10 μg of RNase A (Thermo Fisher) or 125 mM NaCl at 37°C for 1 h. Samples were prepared for native PAGE and SDS-PAGE.

### Native PAGE, SDS-PAGE, phosphorimaging, and immunoblotting.

Samples for native PAGE were diluted in 4× native PAGE sample buffer (Thermo Fisher) and electrophoresed in 4% to 16% native PAGE bis-Tris acrylamide gels (Thermo Fisher) using the Blue native PAGE Novex bis-Tris gel system (Thermo Fisher) at 4°C as described previously (19). Samples for denaturing SDS-PAGE were boiled in SDS-PAGE sample buffer (Bio-Rad) containing β-mercaptoethanol and electrophoresed in 4% to 20% Mini-Protean TGX gels (Bio-Rad).

Polyacrylamide gels containing ^35^S-labeled proteins were fixed with 40% methanol and 10% acetic acid at room temperature (RT) for 30 min, washed with double-distilled water (ddH_2_O), and dried onto filter paper using a gel dryer (Bio-Rad). Dried gels were exposed on a phosphorimaging screen and imaged using a phosphor system scanner (Perkin Elmer, B431200). Band intensities were quantified using ImageJ software.

Polyacrylamide gels containing unlabeled proteins were transferred to nitrocellulose membranes (Bio-Rad) and immunoblotted using the following antibodies: guinea pig σNS-specific polyclonal antiserum (19), chicken μNS-specific polyclonal antiserum (19), and mouse α-tubulin-specific monoclonal antibody (Cell Signaling Technology). IRDye 800CW donkey anti-guinea pig, IRDye 680RD donkey anti-chicken, and IRDye 680LT goat anti-mouse IgG antibodies (Li-Cor) were used as detection reagents. Antibodies were diluted at the following dilutions: 1:1,000 for guinea pig σNS-specific antiserum, 1:5,000 for chicken μNS-specific antiserum, 1:1,000 for mouse α-tubulin-specific antibody, and 1:7,500 for secondary antibodies. Immunoblot images were captured using an Odyssey CLx imaging system (Li-Cor).

### Immunoprecipitation and co-immunoprecipitation assays.

HEK293T cells were transfected with σNS expression plasmids alone or in combination with μNS expression plasmid using FuGene 6 transfection reagent (Promega) at a reagent/DNA ratio of 3:1 in Opti-MEM (Life Technologies). At 24 h posttransfection, cells were lysed with IP lysis buffer (25 mM Tris [pH 7.4], 150 mM NaCl, 1% NP-40 substitute [VWR], 0.5% deoxycholate [DOC], 0.1% SDS) on ice for 30 min or co-IP buffer (20 mM Tris [pH 7.5], 137 mM NaCl, 2 mM EDTA, 0.1% NP-40 substitute) at 4°C for 30 min with rotation. Lysis buffers were supplemented with protease inhibitors (Roche, 11873580001) before use. Following lysis, cellular debris was collected by centrifugation at 20,000 × *g* at 4°C for 20 min. Supernatants were incubated with protein G Dynabeads (Thermo Fisher, 10004D) saturated with σNS-specific monoclonal 2A9 (IP [10]) or 3E10 (co-IP [10]) antibodies at 4°C for 4 h with rotation. Antibodies were saturated on Dynabeads according to the manufacturer’s protocol. Dynabeads were washed with cold lysis buffer, and bound proteins were eluted by boiling in SDS-PAGE sample buffer (Bio-Rad) with β-mercaptoethanol for 10 min. Proteins were analyzed by immunoblotting.

### σNS complementation assays.

GFP or σNS siRNA-expressing cells were cultivated in 6-well plates for virus quantification or 8-well cell culture slides for immunofluorescence (Ibidi, 80826; fluorescence *in situ* hybridization: MatTek, CCS-8). Cells were transfected with GFP or σNS expression plasmids using FuGene 6 transfection reagent. At 24 h posttransfection, cells were adsorbed with reovirus at a multiplicity of infection (MOI) of 5 PFU/cell. Following incubation at 37°C for 48 h, the supernatant was collected, and viral titers were determined by plaque assay. Cells cultivated on slides were processed for fluorescence microscopy.

### Immunogold labeling of Tokuyasu cryosections.

HeLa cells were adsorbed with reovirus T1L M1-P208S at an MOI of 1 PFU/cell. Following incubation at 37°C for 14 h, cells were fixed with 4% paraformaldehyde (PFA) in 0.2 M HEPES buffer (pH 7.4) at RT for 2 h. Free aldehyde groups were quenched with 50 mM NH_4_Cl. Cells were removed from the plates with a rubber policeman and pelleted by centrifugation in a 1.5-ml Eppendorf tube. The cell pellet was embedded in 12% gelatin (TAAB Laboratories) in phosphate-buffered saline (PBS), and after solidification, cubes of 1 mm^3^ were cut and infiltrated with 2.3 M sucrose in PBS at 4°C overnight. Cubes were mounted on metal pins and frozen in liquid nitrogen. Thin cryosections were obtained at −120°C using an FC6 cryo-ultramicrotome (Leica Microsystems), collected from the diamond knife into a 1:1 mixture of 2% methylcellulose in H_2_O and 2.3 M sucrose in PBS, and placed after thawing on 200-mesh grids with a carbon-coated Formvar film. Grids were incubated with σNS-specific monoclonal antibody 2F5 (10) diluted 1:200 in saturation buffer (1% bovine serum albumin [BSA] in PBS) at RT for 1 h. Secondary antibody conjugated with 10-nm colloidal gold particles (British Biocell Int.) was diluted 1:50 in saturation buffer, and grids were incubated at RT for 30 min. After labeling, images were captured using a JEOL JEM-1011 transmission electron microscope operating at 100 kV. At least two independent labeling assays were conducted for each experimental condition.

### Factory-like structure assays.

HEK293T σNS siRNA-expressing cells or HEK293T cells were cultivated in 8-well cell culture slides (immunofluorescence, Ibidi; fluorescence *in situ* hybridization, MatTek). Cells were transfected with various combinations of σ3, σNS, and μNS expression plasmids using FuGene 6 transfection reagent. For combinations of fewer than three plasmids, empty pcDNA3.1+ plasmid was added to the transfection mixtures to maintain identical DNA concentrations for all conditions. At 24 to 48 h posttransfection, cells were processed for fluorescence microscopy.

### Immunofluorescence assays.

Cells were fixed with 4% paraformaldehyde (PFA) diluted in PBS at RT for 30 min, permeabilized in 1% Triton X-100 in PBS at RT for 10 min, and blocked with PBS containing 0.5% bovine serum albumin, 0.1% glycine, 0.05% Tween 20 (PBS-BGT) at 37°C for 10 min. Cells were incubated with σNS-specific monoclonal antibody 3E10 and chicken μNS-specific polyclonal antiserum diluted in PBS-BGT at RT for 1 h, washed with PBS-BGT, probed with species-specific secondary antibodies conjugated with Alexa Fluor 488 or 647 (Thermo Fisher), and counterstained with 4′,6-diamidino-2-phenylindole (DAPI; Invitrogen) to label nuclei. Cells were washed with PBS-BGT and stored in PBS. Antibodies were diluted 1:1,000 for antibody 3E10, 1:1,000 for μNS-specific antiserum, and 1:1,000 for secondary antibodies.

Cell images were captured using a Leica SP8 laser scanning confocal microscope equipped with a 63× oil lens objective. Images were processed and analyzed using ImageJ software with the Fiji processing package. The brightness of each channel was adjusted to that of the appropriate mock signals and normalized for all experimental conditions. The percentage of σNS immunofluorescence signal intensities in factories and factory-like structures was calculated by marking high-intensity μNS immunofluorescence as regions of interest (ROIs). Total σNS immunofluorescence signal intensities within all ROIs of a single cell were determined and then divided by the total σNS immunofluorescence signal intensities detected within the cytoplasm.

### Fluorescence *in situ* hybridization microscopy.

Cells were fixed with 3% PFA diluted in PBS for 30 min and permeabilized in 70% ethanol (EtOH) at 4°C overnight. Cells were rehydrated with wash buffer (10% formamide and 2× SSC [1× SSC is 0.15 M NaCl plus 0.015 M sodium citrate] in diethyl pyrocarbonate [DEPC]-treated water) and incubated with hybridization buffer (10% dextran sulfate, 2 mM vanadyl-ribonucleoside complex [NEB], 0.02% UltraPure BSA [Thermo Fisher], 1 mg/ml Escherichia coli tRNA [Sigma], 2× SSC, and 10% formamide in DEPC-treated water) containing a 1:1,000 dilution of chicken μNS-specific antiserum and 100 nM σNS (quasar670) or σ3 (quasar570) mRNA FISH probes (Biosearch Technologies) at 28°C overnight. The mRNA FISH probe sets consisted of at least 20 probes of ∼20 base pairs in length, and individual probes were designed to bind target sequences at a minimum spacing of two nucleotides between probes (Biosearch Technologies). Following hybridization, cells were washed with wash buffer and incubated with anti-chicken Alexa Fluor 488-conjugated antibody (Thermo Fisher) diluted to 1:1,000 in secondary buffer (2 mM vanadyl-ribonucleoside complex, 0.02% RNA-free BSA, 1 mg/ml E. coli tRNA, 2× SSC, and 10% formamide in DEPC-treated water) at RT for 1 h. Cells were washed with wash buffer and counterstained with DAPI. Glass coverslips were mounted on labeled cells using Prolong Diamond antifade mounting medium (Thermo Fisher).

Cell images were captured using a Leica SP8 laser scanning confocal microscope equipped with a 63× oil lens objective. Images were processed and analyzed using ImageJ software with the Fiji processing package. The brightness of each channel was adjusted to that of the appropriate mock signals and normalized for all experimental conditions. The percentage of FISH signal intensities in factories and factory-like structures was calculated by marking high-intensity μNS immunofluorescence as ROIs. Total FISH signal intensities within all the ROIs of a cell were determined and then divided by the total FISH signal intensities detected within the cytoplasm.

### Statistical analysis.

All experiments were conducted with two to three biological replicates. Data are presented as the mean ± standard error of the mean unless otherwise indicated. Ordinary one-way analyses of variance (ANOVAs) were conducted with Dunnett’s multiple-comparison test. All data and statistical analyses were conducted using GraphPad Prism 8 data analysis software.
