# Supplementary material for: Reovirus Nonstructural Protein σNS Recruits Viral RNA to Replication Organelles
Source: mBio. 2021 Jul 6;12(4):e01408-21. doi: 10.1128/mBio.01408-21 (PMC8406312; doi:10.1128/mBio.01408-21)
Supplement: FIG S1 [file mbio.01408-21-sf001.pdf]

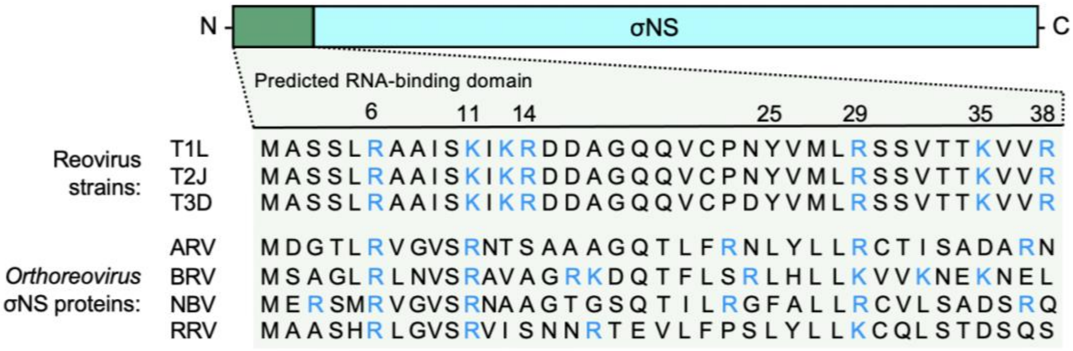

FIG S1 Sequence alignment of the N-terminal 38 amino acids (green bar) of σNS proteins encoded by *Orthoreovirus* species. Shown are σNS sequences from mammalian reovirus strains T1L, T2J, and T3D, avian reovirus (ARV), baboon reovirus (BRV), Nelson Bay orthoreovirus (NBV), and reptilian reovirus (RRV). Positively charged residues are shown in blue.
