# Supplementary material for: Reovirus Nonstructural Protein σNS Recruits Viral RNA to Replication Organelles
Source: mBio. 2021 Jul 6;12(4):e01408-21. doi: 10.1128/mBio.01408-21 (PMC8406312; doi:10.1128/mBio.01408-21)
Supplement: FIG S2 [file mbio.01408-21-sf002.pdf]

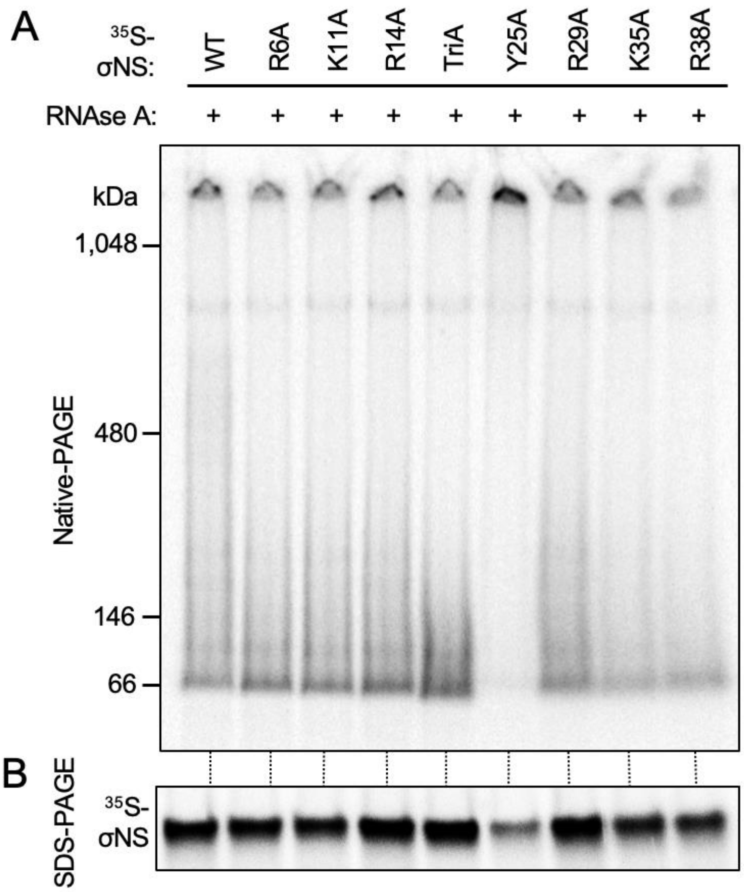

1 **FIG S2** RNase A treatment of σNS mutants disrupt RNA-dependent oligomerization.

2 ^{35}S-labeled σNS was expressed in RRLs and incubated with RNase A. Samples were

3 resolved by (A) native PAGE to preserve oligomeric species or (B) SDS-PAGE to

4 monitor protein expression and visualized by autoradiography.
