# Supplementary material for: Reovirus Nonstructural Protein σNS Recruits Viral RNA to Replication Organelles
Source: mBio. 2021 Jul 6;12(4):e01408-21. doi: 10.1128/mBio.01408-21 (PMC8406312; doi:10.1128/mBio.01408-21)
Supplement: FIG S3 [file mbio.01408-21-sf003.pdf]

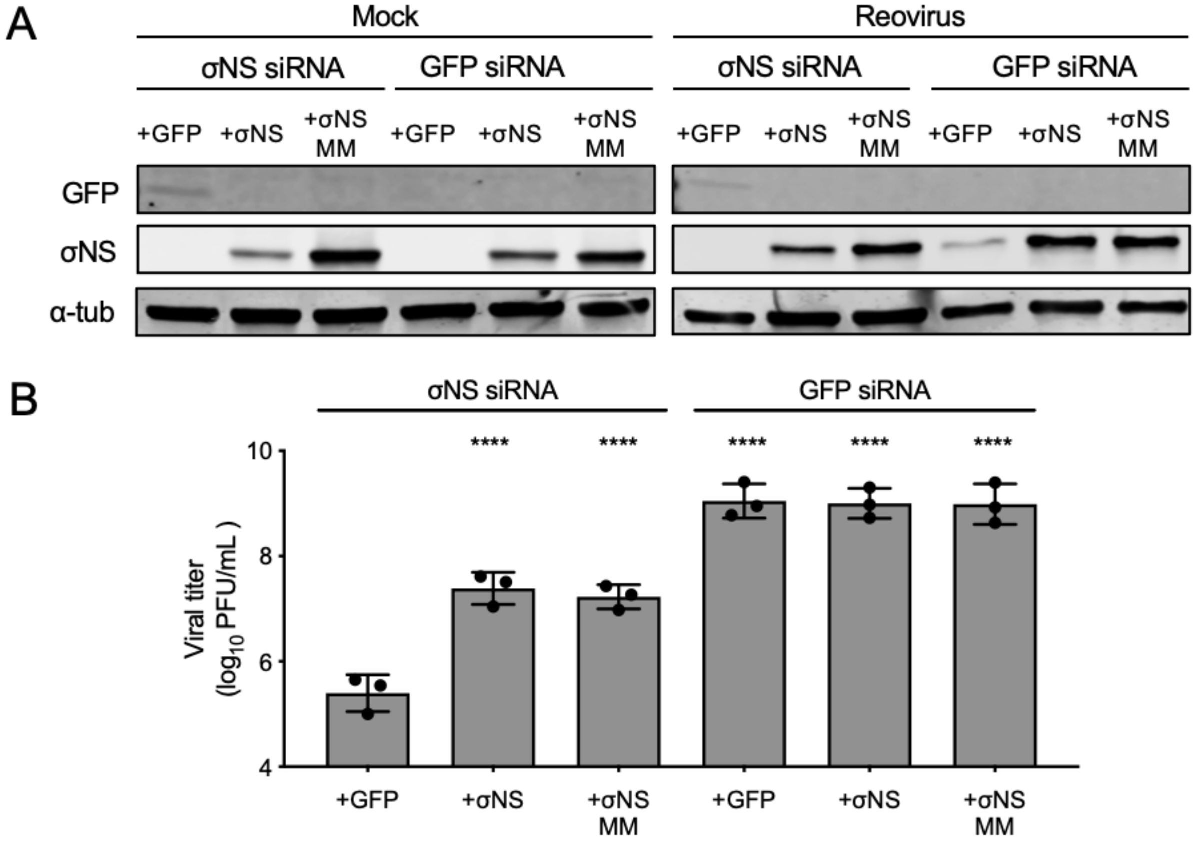

FIG S3 Overexpression of σNS complements siRNA knockdown of σNS during infection. Cells that constitutively express siRNAs directed against σNS or GFP were transfected with expression plasmids encoding GFP, WT σNS (σNS), or WT σNS with σNS-siRNA-resistant sequences (σNS MM) and incubated for 24 h. Cells were adsorbed with reovirus strain T3D at an MOI of 5 PFU/cell and incubated for 24 h. Cell lysates were collected for (A) immunoblotting and (B) infectious virus quantification by plaque assay. (A) Immunoblot analysis of proteins expressed following complementation. Protein expression was evaluated using monoclonal antibodies specific for GFP or alpha-tubulin (α-tub) and guinea-pig sera specific for σNS. (B) Infectious virus quantification following complementation. Titer values that differ

significantly from those obtained from cells expressing siRNAs against σNS complemented with GFP by one-way analysis of variance (ANOVA) with Dunnett's multiple-comparison test are shown (****, $P < 0.0001$).
