# Supplementary material for: Reovirus Nonstructural Protein σNS Recruits Viral RNA to Replication Organelles
Source: mBio. 2021 Jul 6;12(4):e01408-21. doi: 10.1128/mBio.01408-21 (PMC8406312; doi:10.1128/mBio.01408-21)
Supplement: FIG S4 [file mbio.01408-21-sf004.pdf]

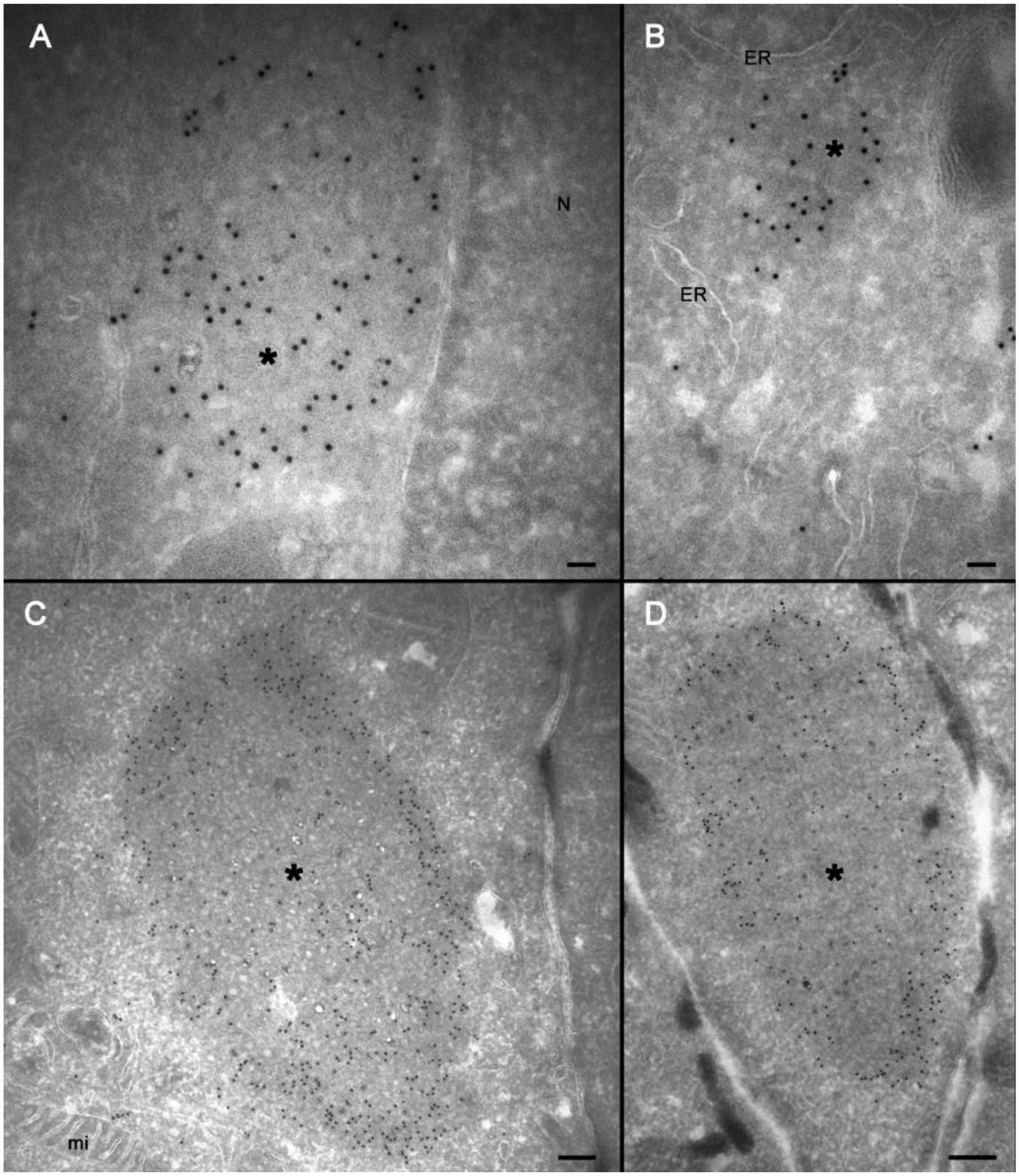

FIG S4 Immunogold labeling of σNS proteins in Tokuyasu cryosections of reovirus-infected cells. Cells were adsorbed with reovirus strain T1L M1 P208S at an MOI of 1 PFU/cell, incubated for 14 h, frozen in liquid nitrogen, and sectioned at -120°C. Thawed cryosections were processed for immunogold labeling using σNS-specific monoclonal

antibody 2F5, followed by a secondary antibody bound to 10 nm colloidal gold spheres. Cryosections were imaged using transmission electron microscopy. Panels A and B show representative images of small, punctate, viral factories, and panels C and D show representative images of larger mature factories. Nucleus (N), endoplasmic reticulum (ER), and mitochondria (mi) are labeled when visible surrounding a viral factory (*). Bars, 50 nm (A and B), 200 nm (C and D).
