# Supplementary material for: Reovirus Nonstructural Protein σNS Recruits Viral RNA to Replication Organelles
Source: mBio. 2021 Jul 6;12(4):e01408-21. doi: 10.1128/mBio.01408-21 (PMC8406312; doi:10.1128/mBio.01408-21)
Supplement: FIG S5 [file mbio.01408-21-sf005.pdf]

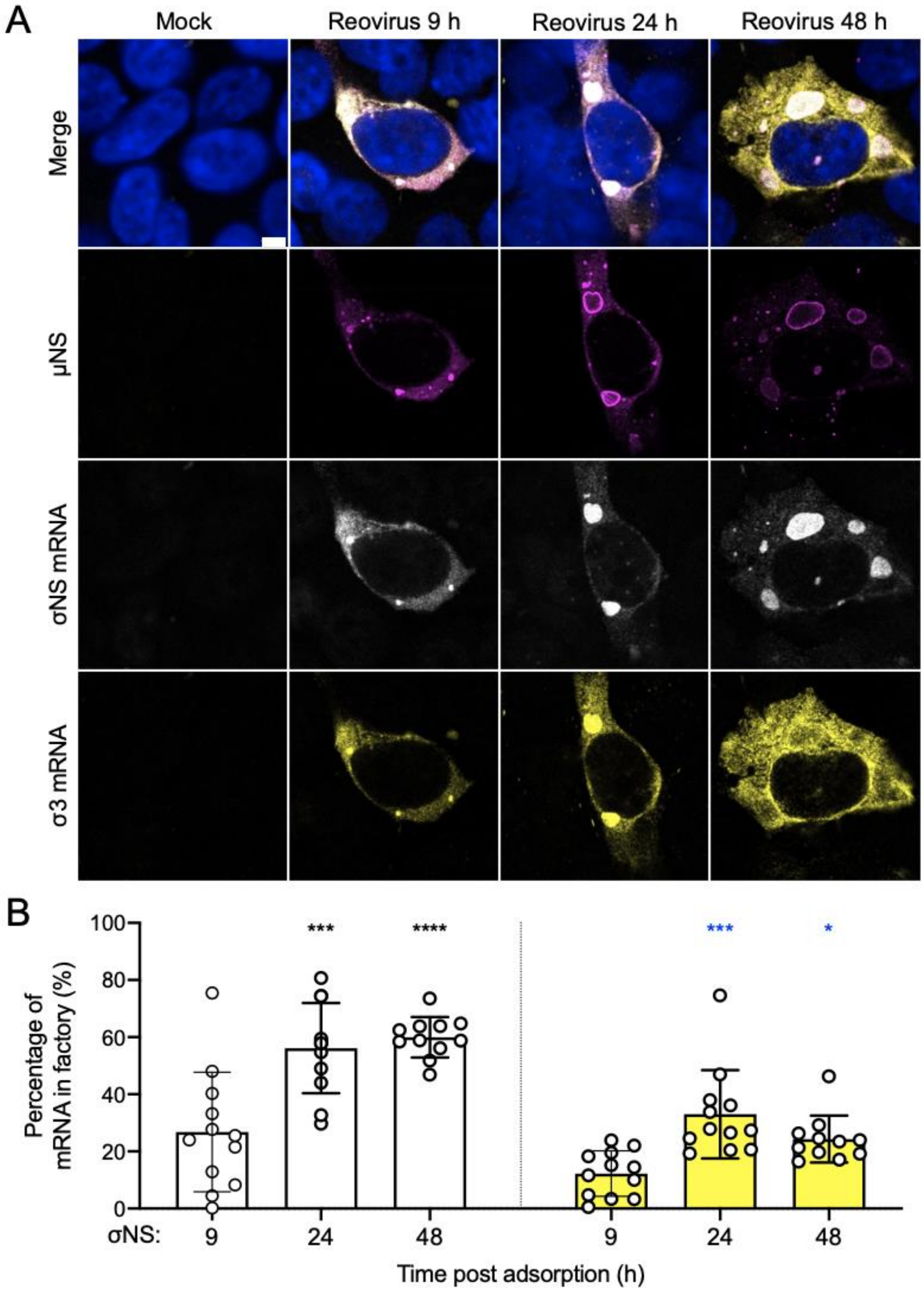

F IG S5 Reovirus transcripts differentially localize in viral factories at late timepoints post-adsorption. HEK293T cells were adsorbed with reovirus strain T3D at an MOI of 20 PFU/cell, incubated for 9, 24, and 48 h, fixed, stained using RNA FISH probes specific

for σNS mRNA (white) or σ3 mRNA (yellow), μNS-specific antiserum (magenta), and DAPI (blue), and imaged using confocal microscopy. Bar, 5 μm. The percentage of cytoplasmic (B, white bars) σNS-mRNA and (B, yellow bars) σ3-mRNA FISH signals in reovirus factories was quantified by dividing the sum of FISH signal in reovirus factories by the sum of cytoplasmic FISH signal. Individual data points represent single cells. Percentage values that differ significantly from those obtained from 9 h post-adsorption by one-way analysis of variance (ANOVA) with Dunnett's multiple-comparison test are shown (*, $P < 0.0332$; ***, $P < 0.0002$; ****, $P < 0.0001$).
