# Supplementary material for: Reovirus Nonstructural Protein σNS Recruits Viral RNA to Replication Organelles
Source: mBio. 2021 Jul 6;12(4):e01408-21. doi: 10.1128/mBio.01408-21 (PMC8406312; doi:10.1128/mBio.01408-21)
Supplement: TABLE S1 [file mbio.01408-21-st001.pdf]

Supplemental Table 1

	Sequence (5′→3′)	
	**Forward (F)**	**Reverse (R)**
T3D M3 5’-KpnI-NotI-3’	CGACGGTACCATGGCTTCATTCAAGGGATTCTCCG	ATCACAGGCGGCCGCTTACAACTCATCAGTTGGAACAGAGAAATC
T3D S3 R6A	CTTGGAGATCGCAGCTGCGAGTGAGGAAGCCATG	CATGGCTTCCTCACTCGCAGCTGCGATCTCCAAG
T3D S3 K11A	CGTCATCCCTCTTGATCGCGGAGATCGCAGCTCTGA	TCAGAGCTGCGATCTCCGCGATCAAGAGGGATGACG
T3D S3 R14A	GCTGACCGACGTCATCCGCCTTGATCTTGGAGATCG	CGATCTCCAAGATCAAGGCGGATGACGTCGGTCAGC
T3D S3 TriA	CACTCAGAGCTGCGATCTCCGCGATCGCGGCGGATGACGTCGGTCAGCAAG	CTTGCTGACCGACGTCATCCGCCGCGATCGCGGAGATCGCAGCTCTGAGTG
T3D S3 Y25A	CGTCGGTCAGCAAGTTTGTCCTAATGCTGTCATGCTGCGG	CCGCAGCATGACAGCATTAGGACAAACTTGCTGACCGACG
T3D S3 R29A	CTTTGTTGTGACAGAGGACGCCAGCATGACATAATTAGGA	TCCTAATTATGTCATGCTGGCGTCCTCTGTCACAACAAAG
T3D S3 K35A	ACATTTCGTACCACCGCTGTTGTGACAGAGGACCGCAG	CTGCGGTCCTCTGTCACAACAGCGGTGGTACGAAATGT
T3D S3 R38A	AATTTGATACTCAACCACATTTGCTACCACCTTTGTTGTGACAGAG	CTCTGTCACAACAAAGGTGGTAGCAAATGTGGTTGAGTATCAAATT
